# Parallel repair mechanisms in plants and animals

**DOI:** 10.1242/dmm.049801

**Published:** 2023-01-27

**Authors:** Timothy C. Byatt, Paul Martin

**Affiliations:** School of Biochemistry, University of Bristol, University Walk, Bristol BS8 1TD, UK

## Abstract

All organisms have acquired mechanisms for repairing themselves after accidents or lucky escape from predators, but how analogous are these mechanisms across phyla? Plants and animals are distant relatives in the tree of life, but both need to be able to efficiently repair themselves, or they will perish. Both have an outer epidermal barrier layer and a circulatory system that they must protect from infection. However, plant cells are immotile with rigid cell walls, so they cannot raise an animal-like immune response or move away from the insult, as animals can. Here, we discuss the parallel strategies and signalling pathways used by plants and animals to heal their tissues, as well as key differences. A more comprehensive understanding of these parallels and differences could highlight potential avenues to enhance healing of patients’ wounds in the clinic and, in a reciprocal way, for developing novel alternatives to agricultural pesticides.

## Teaser

**Summary:** The biology underpinning wound repair in plants and animals is very different, but a search for parallels reveals several shared signals and analogous mechanisms that may offer valuable lessons across the two kingdoms.

## The first step is perceiving the wound

**Fig. 1.**
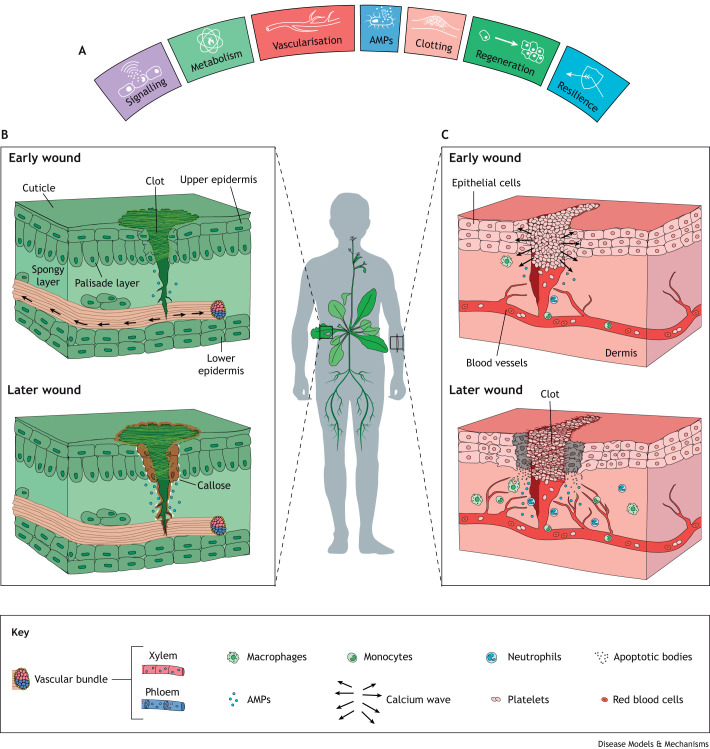
**The shared hallmarks of wound repair mirrored across the plant and animal kingdoms.** Many wound-response processes have parallels across the animal and plant kingdoms. (A) Here, we present seven hallmarks [signalling, metabolism, vascularisation, antimicrobial peptides (AMPs), clotting, regeneration, resilience], that share some degree of similarity across the phyla. (B,C) The schematic illustrates wounds in a plant (*Arabidopsis thaliana*) versus a human, and the higher-magnification views to the left and right, respectively, show some of the hallmark stages within the two repair processes. (B) A higher-magnification view of a cross-section of the damaged leaf at early and later stages of wound healing. In the early stage of wound healing, the calcium wave (black arrows) permeates from the wound-edge cells and travels along the vascular bundle, made up of the xylem and phloem. To prevent pathogen entry, a semi-permanent ‘clot’ is generated from sap and phloem proteins that reversibly form disulphide cross-links to generate a polymer. Some antimicrobial peptides are released by cells in the wound area to protect against infection. In later stages of wound healing, neighbouring dead wound-edge cells have been reinforced by lignification (brown) and callose formation (white). More antimicrobial peptides are also released to further protect against infection. (C) A higher-magnification view of a cross-section of the human skin wound at early and later stages of wound healing. In the early stage of wound healing, the calcium wave (black arrows) rapidly permeates from the wound-edge cells but remains localised at the wound site. A wound inflammatory response is also triggered, which includes the release of antimicrobial peptides and the recruitment of motile innate immune cells, to clear pathogens. Alongside these processes, a clot is formed of activated platelets to plug the breach in the damaged epithelial cell layer. In later stages of wound-healing responses, cells at the wound edge undergo apoptosis (grey) and release apoptotic bodies. Neighbouring epithelial cells proliferate and collectively migrate to reform the damaged barrier. Increased levels of antimicrobial peptides are released alongside a greater number of macrophages that patrol the wound site, digesting cell and matrix debris and releasing attractants and signalling molecules. Circulating neutrophils and monocytes extravasate from vessels and are drawn towards the wound by chemotactic signalling molecules. New blood vessels sprout towards the wound in response to growth factors. Furthermore, the temporary clot is reinforced with a fibrin mesh to fully seal the wound.

Wounding has a multitude of causes, from a predator's jaws to a burn in the home or an experimental laser in the laboratory. Tissues adjacent to the wound perceive this damage and then must determine the extent of the danger, for example whether the wound is infected, in order to respond appropriately (Fig. 1).

**Fig. 2.**
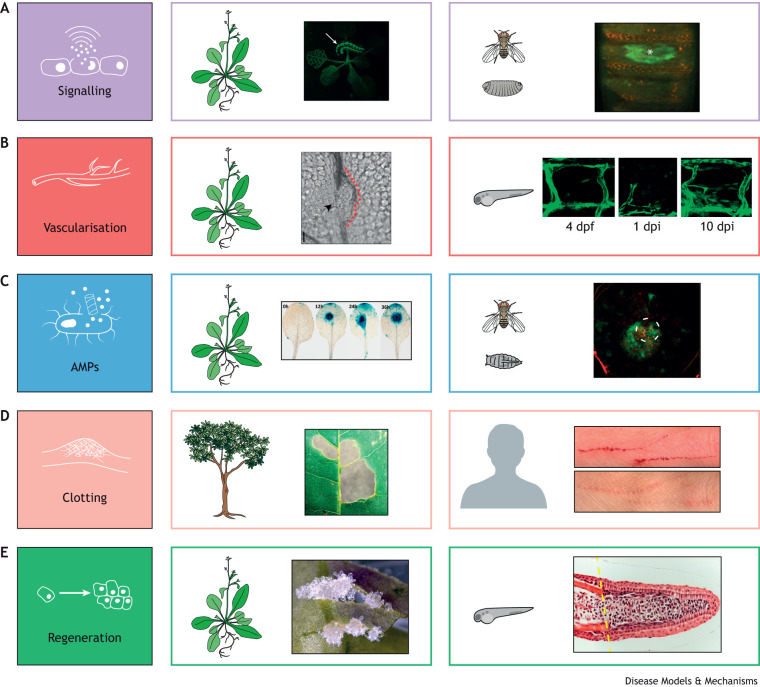
**Examples of parallel hallmarks.** Examples of the shared wound-response hallmarks across the two phyla. (A) Signalling: a calcium wave [revealed by GCaMP fluorescent (green) reporters] spreading through the vasculature of *Arabidopsis thaliana* (left) in response to wounding from a feeding caterpillar (arrow) (Nguyen et al., 2018). In a *Drosophila* embryo (right), a similar calcium wave (green) is triggered in response to an experimental laser wound (asterisk indicates wound centre) (Razzell et al., 2013). (B) Vascularisation: after wounding of an Arabidopsis leaf (left), a new vessel forms (red dotted line), bypassing the site of the previously damaged vessel (arrowhead) (Radhakrishnan et al., 2021). In a transgenic zebrafish (right), GFP-Fli reveals the flank square vascular blocks and how these undergo neoangiogenesis after wounding (Gurevich et al., 2018). dpf, days post fertilization; dpi, days post injury. (C) Antimicrobial peptides (AMPs): Arabidopsis leaves (left) expressing the AMP LTPg5 (blue) in response to infection visualised using a GUS reporter line. *Drosophila* (right) fat body cells (red) expressing and delivering the AMP Attacin (green) at the site of a small laser wound (white dashed line circle). (D) Clotting: a damaged leaf (left) with numerous lignified patches (brown), sealing sites of insect predation damage. Similarly, the bleeding from a scratch wound to a human finger (right) is stemmed by clotting and subsequently a scab forms to act as a temporary patch over the skin wound until it heals. (E) Regeneration: pluripotent callus cells (white) proliferating at the margins of a leaf wound (left) (Ikeuchi et al., 2013). Histology of a regenerating zebrafish tail blastema (right), 3 days after amputation (at the yellow dashed line), with proliferating cells that will go on to replace the missing tissue (Chablais and Jaźwińska, 2010). Image permissions: (A) The Arabidopsis image was reproduced with the authors' permission from Nguyen et al. (2018) under the terms of the CC-BY-NC-ND licence. This image is not reproduced under the terms of the CC-BY licence of this article. For permission to reuse, please see Nguyen et al. (2018). The *Drosophila* image was reproduced with the authors' permission from Razzell et al. (2013) under the terms of the CC-BY 3.0 licence. (B) The Arabidopsis image was reproduced with the authors' permission from Radhakrishnan et al. (2021). This image is not reproduced under the terms of the CC-BY licence of this article. For permission to reuse, please see Radhakrishnan et al. (2021). The zebrafish image was reproduced with the authors' permission from Gurevich et al. (2018) under the terms of the CC-BY 4.0 licence. (C) The Arabidopsis and *Drosophila* images were reproduced with the authors' permission from Ali et al. (2020) and Franz et al. (2018), respectively, under the terms of the CC-BY 4.0 licence. (D) Stephanie Foster granted us permission to use the images of her wounded hand. (E) The Arabidopsis image was reproduced with the authors' permission from Ikeuchi et al. (2013) under the terms of the CC-BY 3.0 licence. The zebrafish image was reproduced with the authors’ permission from Chablais and Jaźwińska (2010). This image is not published under the terms of the CC-BY licence of this article. For permission to reuse, please see Chablais and Jaźwińska (2010).

In both plants and animals, wound activation begins with a flash of calcium signalling that radiates from the site of the mechanical insult. This first signal can be visualised using GCaMP reporter (see Glossary, Box 1) transgenes, enabling live imaging of the calcium wave in, almost, real time (Fig. 2A) (Nguyen et al., 2018; Razzell et al., 2013; Xu and Chisholm, 2011; Toyota et al., 2018). For example, after wounding an Arabidopsis leaf, a calcium wave is initiated within 2 s and spreads at a speed of ∼1 cm/min until it reaches a vein, from where it is systemically transmitted, through the vascular system, to the distal extremities of the plant (Toyota et al., 2018). In animal tissues, the calcium flash is even more instantaneous, but remains more local (Razzell et al., 2013; Xu and Chisholm, 2011).

**Box 1. Glossary**

**Antimicrobial peptides (AMPs):** small peptides with diverse mechanisms for host defence, including broad-spectrum antibacterial, antiparasitic, antifungal and antiviral activities.

**Apoptosis:** a mechanism of programmed cell death found in multicellular organisms that is used to clear unwanted or significantly damaged cells during development and repair.

**Autotomy:** self-amputation of a limb or tissue to escape danger.

**Auxin:** a phytohormone class involved in many plant growth and development processes, as well as the wound response.

**Blastema:** a mass of dedifferentiated cells that proliferate and subsequently re-differentiate to repair lost limbs, organs or tissues in regeneration-competent animals.

**Callose:** a polysaccharide that creates a barrier in various plant processes, for example lining the pores of sieve elements, or is deposited as part of the plant response to infection and wounding.

**GCaMP reporter:** a fusion protein of green fluorescent protein, calmodulin and M13 from myosin light-chain kinase. This fusion protein fluoresces green when calcium is bound, therefore enabling live imaging of calcium dynamics in transgenic organisms.

**G protein-coupled receptors:** transmembrane receptors that initiate a cellular process in response to the binding of a ligand, such as ATP or epinephrine.

**Jasmonate:** a phytohormone important for plant signalling, abiotic and biotic stress in plants. Activation of jasmonate signalling triggers the transcription of many wound and defence genes.

**Phloem:** one of the two plant vessels that form the vascular bundle (see ‘xylem’). The phloem is a tubular structure on the outside of the vascular bundle that transports sugars, amino acids and water bidirectionally  from areas of high concentration to areas of low concentration.

**Sieve element:** a specialised cell with only the primary cell wall that forms part of the phloem. The cells have a sieve-like area with pores through the cell wall, enabling the conduction of dissolved sugars and amino acids through the phloem.

**Trichome:** hair-like structures that protrude from the surface of the leaves and stem of some plant species. Adapted as a physical deterrent to herbivores and linked to mechanosensory systems.

**Xylem:** one of the two plant vessels that form the vascular bundle (see ‘phloem’). The xylem is a tubular structure reinforced by lignin that transports water and minerals unidirectionally from the roots up the stem of the plant drawn by the transpiration stream.

Damage-triggered calcium waves are transduced through various mechanisms in both plants and animals. Extracellular calcium enters through microtears in the plasma membrane of damaged cells and, in animals, spreads through gap junctions to neighbouring cells (Shannon et al., 2017). Presumably, a similar process occurs via the plasmodesmata channels linking neighbouring plant cells. However, there are also less direct routes for the calcium wave. For example, a study in *Drosophila* revealed how proteases released from damaged cells cleave and activate extracellular growth-blocking peptides that are detected by G protein-coupled receptors (Box 1), such as Methuselah-like 10, on cells neighbouring the wound edge, leading to calcium release from their endoplasmic reticulum (O'Connor et al., 2021). In plants, a parallel signalling cascade exists with glutamate receptor-like (GLR) channels regulating calcium influx when glutamate leaks from the ruptured phloem (Box 1) and binds to GLRs on the xylem contact cells (Box 1) and phloem sieve elements (Box 1) (Qi et al., 2006; Nguyen et al., 2018; Mousavi et al., 2013; Toyota et al., 2018). Calcium-dependent proteases then cleave pro-plant elicitor peptides (PEPs) into active forms that bind neighbouring cells’ PEP receptors to trigger innate immune responses (Hander et al., 2019).

## Wound signalling cascades in life and death

In animal tissues, the calcium wave initiates release of permissive immune cell attractants, including reactive oxygen species (ROS), in particular H_2_O_2_, which are produced by NADPH oxidase enzymes (Bedard and Krause, 2007). In *Drosophila*, Duox, the NADPH oxidase enzyme responsible for generating H_2_O_2_, is directly responsive to calcium (Razzell et al., 2013). Similarly, in plants, transmembrane NAPDH oxidases, called respiratory burst oxidase homologues (RBOHs), produce H_2_O_2_ in response to wounding, which is regulated by calcium-dependent phosphorylation or by direct binding of calcium to RBOH domains (Dubiella et al., 2013; Drerup et al., 2013). Is this calcium-dependent ROS release conserved from a common plant and animal ancestor, or the result of convergent evolution? We presume the former because calcium-regulated NAPDH oxidases are present in the most ancient organisms (Kawahara et al., 2007), and all examined so far exhibit a wound activated calcium/ROS axis (Moon et al., 2022).

ROS production in both plants and animals can function directly as a microbicidal but also as a signalling mechanism to activate various elements of the defence response at the wound site (Mhamdi et al., 2010; Chang et al., 2004). Crucial to organism survival, ROS initiate key mechanisms of the wound repair cascade. One such mechanism is apoptosis (Box 1), which is critical for driving proliferation as its inhibition in hydra and *Xenopus* eliminates their regenerative response (Tseng et al., 2007; Galliot and Chera, 2010). H_2_O_2_ release at the wound site triggers apoptosis, and inhibition of ROS-producing enzymes also inhibits regeneration (Love et al., 2013; Tobiume et al., 2001). Studies in *Xenopus* and hydra have shown that ROS-triggered apoptosis, and subsequent proliferation, is mediated by WNT/β-catenin signalling (Galliot and Chera, 2010; Love et al., 2013). During fin regeneration in adult zebrafish, it seems that ROS production leads to a second, later wave of apoptosis that, in turn, is key to formation of the regenerative blastema (Box 1) (Gauron et al., 2013). In some animals, successful regeneration can be potentiated by sacrificing entire stretches of damaged tissue, involving programmed cell death or autotomy (Box 1) along pre-defined fracture planes (Box 2).

**Box 2. Sacrificing cells and tissues for the greater good of the organism**

In the animal world, the most efficient option when faced with a predator is to flee, which of course is not possible for a plant. A dramatic, alternative strategy adopted by some animals involves active tissue sacrifice. This process, termed autotomy, has examples across all phyla, including crustacea claw shedding and lizards losing their tails, and even the African spiny mouse that appears to have developed mechanisms for shedding, and subsequently regenerating, without scarring, large amounts of skin when attacked (Seifert et al., 2012; Maginnis, 2006).

Because many plants lose their leaves seasonally, it would seem that shedding an injured leaf would be an effective strategy to prevent more general predation or infection. However, although there are some notable examples, such as the Bermuda buttercup, the leaves of which are ‘dropped’ during predation (Shtein et al., 2019), autotomy appears to be a surprisingly underused defence strategy by plants.

Instead, plants routinely sacrifice wound-edge cells as part of their damage response. Following injury, a band of cells only a few rows proximal to the damaged site undergoes programmed cell death, accompanied by cell wall reinforcement with phenolic compounds, such as lignin, to generate a barrier against infection (Chezem et al., 2017; Vance et al., 1980). This lignified barrier is then further reinforced by the deposition of suberin (a polyester of fatty acids and glycerol) to provide further resistance to degradation, and protection against pathogen entry (Fig. 2D) (Serra and Geldner, 2022). This process is triggered downstream of immediate wound signals, including reactive oxygen species and abscisic acid, and is tightly regulated to prevent the domain of death extending back into the healthy tissue. MYB108 is a jasmonate-inducible transcription factor, which negatively regulates abscisic acid biosynthesis, and if MYB108 mutant plants are wounded, then a cascading wave of cell death is triggered that can kill the whole organism (Cui et al., 2013). Equivalent wound inhibitory signals have barely been investigated in vertebrate animal models, but a very informative screen of regulators of wound-activated transcription in *Drosophila* has revealed several genes, including *Flo2* and *Src42a*, that appear to be required to limit the spread of early wound signals (Juarez et al., 2011). These endogenous ‘dampening’ mechanisms might prove to be potential therapeutic targets to activate in order to limit the extent of negative aspects of wound healing, such as fibrosis; or conversely, to switch off to prolong and extend more positive wound cell behaviours, such as re-epithelialisation, that will speed up healing.

There is also an undoubtedly important role for cell death in the plant wound response. Recent studies in Arabidopsis have shown that cell death leads to expression of the transcription factor ethylene-responsive factor 115 (ERF115) in cells neighbouring the dying epidermal wound-edge cells (Johnson et al., 2018). Subsequently, ERF115 triggers regenerative cell division (Heyman et al., 2016), and thus may represent a parallel axis of regenerative control to the animal WNT/β-catenin pathway because of its induction by cell death (Zhou et al., 2019; Kong et al., 2018). Although cell death is clearly an important activator, it is crucial to limit its spread using ‘dampening’ mechanisms to protect the whole organism (Box 2).

“These adjustments in metabolic strategies in response to wounding appear to be essential, across phyla […] A better understanding of their activation may lead to development of clinical therapeutics and agricultural treatments.”

## Altering metabolism at the wound site

ROS signalling is also fundamental for wound-induced changes in metabolism, in both animals (Love et al., 2014) and plants (Jacobo-Velazquez et al., 2015). The metabolic demands at a site of damage and repair are hugely increased to drive cell proliferation and other repair processes. Just as in rapidly growing cancer where the Warburg effect switches metabolic pathways from the citric acid cycle and oxidative phosphorylation to anaerobic glycolysis, it seems that injury and repair can also lead to a metabolic shift in cells (Sinclair et al., 2021; Scott et al., 2022).

Several decades ago, Thomas Hunt and colleagues revealed high levels of lactate in healing wounds in rabbits and speculated that switching to a glycolytic pathway might be key to some elements of the repair process (Ghani et al., 2003). Gene expression studies in the regenerating *Xenopus* tadpole limb show that many genes linked to glycolytic metabolism are locally induced here too (Love et al., 2014). More recent single-cell transcriptomic analysis of mouse skin wounding indicates a dramatic alteration in expression of metabolism-associated genes (Haensel et al., 2020). In this study, genes associated with oxidative phosphorylation were downregulated, and glycolysis-associated genes were upregulated, in sub-populations of wound-edge cells.

The plant wound response also leads to alterations in metabolism. A recent proteomics study revealed how wounding leads to an increase in starch and sucrose metabolism, likely providing the carbon for biosynthesis of amino acids and secondary metabolites (Guan et al., 2021).

These adjustments in metabolic strategies in response to wounding appear to be essential, across phyla, to provide the energy and resources required to repair a wound and prevent infection at the site of injury. A better understanding of their activation may lead to development of clinical therapeutics and agricultural treatments, or potential prognostic indicators of the ‘health’ status of healing tissues.

## Mechanisms for combatting infection

In all organisms, a breach in any barrier layer will result in invasion by pathogens, including bacteria, fungi and viruses. These pathogens must be rapidly quelled, or they will overcome the organism.

In all multicellular organisms, these invaders are recognised by pattern recognition receptors (PRRs), of which the most prominent in animals are the Toll-like receptors (Qian and Cao, 2013). In most animal species, there exists some degree of an immune system that has evolved to recognise and clear pathogens when the barrier layer becomes damaged and infection occurs. This triggers a wound inflammatory response, which, at early stages, is largely dependent on motile innate immune cells, such as neutrophils, monocytes and macrophages. Only if wounds become chronic and/or very infected, will the adaptive immune system be enlisted too (Weavers and Martin, 2020).

Plants have a very different portfolio of PRRs (Noman et al., 2019) and do not possess motile immune cell lineages. However, there is a more primitive component to the inflammatory response that is shared between animals and plants, which involves the upregulation and release of antimicrobial peptides (AMPs; Box 1; Fig. 2C). AMPs are crucial to plant immunity. Some AMPs are constitutively expressed, but others are locally or systemically upregulated in response to jasmonate (Box 1) synthesis, downstream of the immediate wound signals (Browse, 2009; Howe et al., 2018; Lenglet et al., 2017). The largest family of AMPs in plants are the defensins, with over 300 members identified in Arabidopsis (Silverstein et al., 2005). Defensins exhibit an impressive range of functions, from pathogenic cell membrane disruption to inhibition of predatory digestive enzymes (Kovaleva et al., 2020). In humans, over 30 beta-defensins have been identified (Schutte et al., 2002), and these appear to act through similar mechanisms (Pazgier et al., 2006). Plant and invertebrate defensins share a characteristic cysteine-stabilised alpha helix, and all defensins, including mammalian defensins, share a similarly sized triple-stranded antiparallel beta sheet, suggesting that defensins are an evolutionarily conserved class of AMPs across kingdoms (Broekaert et al., 1995). Historically, AMPs have somewhat slipped under the radar in animal repair studies, but, increasingly, they are being investigated as important small-molecule candidates for multiple medical applications associated with tissue repair and tumour progression (Baindara et al., 2017; Sun et al.; Williams et al., 2018; Mangoni et al., 2016).

## Sealing the wound site with a ‘clot’

One of the most urgent tasks at any site of tissue damage is to rapidly seal the gap to prevent loss of tissue fluids and influx of infectious agents. When a vertebrate animal is wounded, platelets or their equivalents leak from ruptured blood vessels adjacent to the wound site, become activated and degranulate upon exposure to extravascular factors, including collagen (Ivanciu and Stalker, 2015). Simultaneously, thrombin cleaves fibrin to fibrinogen to form a cross-linked mesh at the wound site, and the platelets become embedded in this mesh, forming a clot (Martin, 1997; Jansen and Hartmann, 2021; Li et al., 2010). In insects, a similar localised release of vesicular granules and formation of a coagulatory fibrillar network occurs, although there are no platelet equivalents and few, if any, molecular parallels to vertebrate clotting (Schmid et al., 2019).

An analogous process occurs at the site of injury in plants. As plant vessels are ruptured, sap exudes from their cut ends, and phloem proteins reversibly form disulphide cross-links to generate a polymer analogous to the fibrin mesh in a vertebrate animal clot (Ernst et al., 2012; Kehr, 2006). This initial ‘clot’ can then be reinforced by callose (Box 1) deposition to prevent pathogen entry. In plants, this ‘clot’ is a semi-permanent fixture, in contrast to the temporary protective scab/clot formed in certain animals, which is subsequently degraded and shed as the epidermis repairs.

In an example of parallel co-evolution, sap-sucking aphids have evolved to circumvent plant ‘coagulatory’ defences. Much like mosquitos injecting anticoagulants when sucking blood, aphids inject proteases, thereby preventing plugging of the sieve element allowing them to feed for extended periods (Walling, 2000; Furch et al., 2015). However, interestingly, aphids can also aid plant healing, as soldier aphids have been observed depositing body fluids that harden to form protective ‘scabs’ on leaves wherever they are feeding, in order to prevent their host's inconvenient early death (Kutsukake et al., 2009).

The plugging of a clot to seal the wound site, alongside an immune response in animals and release of AMPs in plants and animals, provides the time required to restore a barrier layer and rebuild missing tissues beneath the seal (Fig. 2B).

“We need to be looking to plants – just as much as to axolotls – to identify the best epigenetic clues to enable better regenerative capacity in our own tissues and organs following damage.”

## Regeneration versus scarring

Among the animal kingdom, there is great disparity in the regenerative capacity of different species after injury. This ranges from healing with a scar in adult mammalian tissues (Fig. 2D), healing without a scar in mammalian embryos (Ferguson and O'Kane, 2004), and ultimately to being able to regenerate whole organs in planaria flatworms, and many fish and amphibian species (Tanaka and Reddien, 2011).

Studies in zebrafish and axolotl show how cells proximal to the injury site form a regenerative mass of dedifferentiated, multipotent stem-like cells, collectively called a blastema (Blum and Begemann, 2012; McCusker et al., 2015). Blastemal formation is regulated by retinoic acid and growth factor signals, key amongst these being FGF and WNT/β-catenin signalling, which drive cell cycle re-entry (Wehner and Weidinger, 2015). Blastemal cells will then re-differentiate, recapitulating developmental pathways to replace the missing cells and tissues of the damaged organ. However, regeneration-competent animals are limited by cellular memory. Within the blastema of an axolotl limb, cells remain lineage and tissue specific, and re-differentiate into their original cell type (Kragl et al., 2009). This means a tissue cannot be regenerated if it has been entirely lost in the wound. Plants, by contrast, are much more widely competent at regenerating their lost tissues. Their equivalent of a blastema is a callus that exhibits much greater plasticity during regeneration (Fig. 2E), and their capacity for trans-differentiation enables regeneration of any tissue after predation, which explains why plants can be propagated from cuttings (Ikeuchi et al., 2016). However, although plants have the capacity to regenerate a leaf, or even a whole plant from a cutting, they tend not to replace small patches of missing tissue lost – for example, within a wounded leaf – which is perhaps due to inhibitory feedback from cell wall biogenesis at the wound site.  The mechanisms behind these different context-dependent responses to wounding are still not fully understood (Ikeuchi et al., 2013).

Epigenetic modification lies at the heart of regeneration competency in animals, with histone modifications suppressing or activating developmental pathways required for regeneration, alongside retention of lineage identity to prevent aberrant homeotic transformation (Hayashi et al., 2020). Studies on zebrafish fin regeneration have revealed rapid changes in histone modification, in particular loss of polycomb-deposited histone H3K27me3 repressive marks on the promoters of key growth and proliferation genes, which are pivotal at early stages in the regenerative process (Stewart et al., 2009). It seems that, as well as clearing repressive histone marks, blastemal cells need to reduce levels of DNA methylation in order to re-express some developmental genes that enable proliferation and other regenerative activities (Takayama et al., 2014). To a lesser degree, some polycomb-mediated epigenetic changes to histone marks also occur in less regenerative organisms, such as mice. This enables wound-responsive changes in cell migration and proliferation, as well as partial epithelial–mesenchymal transition that is needed to enable wound re-epithelialisation (Shaw and Martin, 2009).

In plants, cellular identity is, in part, also maintained in differentiated cells by polycomb-deposited H3K27me3 marks, and loss of these marks is critical to the dedifferentiation required for callus formation following wounding (Mozgova et al., 2017; Ikeuchi et al., 2015). The leaf to callus transition requires repression of leaf regulatory genes by histone trimethylation, in parallel with the demethylation of the auxin (Box 1) hormone pathway and root regulatory genes (He et al., 2012). Another key epigenetic marker of wound-induced transcriptional activation of plant genes is histone 3 acetylation, as inhibition of histone acyltransferases abolishes wound callus formation (Rymen et al., 2019). Interestingly, many rapidly wound-induced genes are pre-primed for transcription with acetylation marks, poising them for induction by wound-activated transcriptional regulators (Rymen et al., 2019). It may be that the plant genome is more effectively cleansed of inhibitory epigenetic marks upon wounding, making it more plastic in the regenerative phase. Therefore, we need to be looking to plants – just as much as to axolotls – to identify the best epigenetic clues to enable better regenerative capacity in our own tissues and organs following damage.

“As we better understand these natural resilience mechanisms, more advanced treatments might be developed to mimic or activate these systemic resistance pathways to improve crop survival, and some of these strategies may also be ‘borrowed’ for use in the clinic.”

## Priming tissue resilience

Although all organisms have developed strategies for repair, preventing damage in the first place by shielding or protecting tissue is clearly advantageous and will also have been evolutionarily selected for wherever the cost is not too great. Permanent examples of this include camouflage or mimicry, or obnoxious taste, but there are also more transient resilience mechanisms that prime tissues to minimise damage. Recent studies in flies and mice have shown a series of signalling pathways activated by damage itself, and by the associated inflammatory response, which triggers local upregulation of enzymes that sequester ROS, as well as upregulation of DNA and protein repair machineries (Weavers et al., 2019; Haertel et al., 2014; Telorack et al., 2016). These mechanisms that transiently enhance tissue repair capability would be extremely useful to harness in the clinic, for example prior to elective surgery, and might also be beneficial to activate in crops prior to times of potential infestation. However, whether plants have equivalent ‘resilience’ mechanisms remains to be addressed.

Plants clearly have mechanical strategies for detecting impending attack, utilising their sensory trichomes (Box 1), which, when disturbed, can lead to upregulation of antipredator toxins before the first bite (Zhou et al., 2017; Peiffer et al., 2009). Moreover, plants exhibit priming of defence pathways in response to sequential stimuli, with damage or even soft mechanical stress leading to localised disease resistance (Benikhlef et al., 2013; Chassot et al., 2008). Prior exposure to pathogens has powerful priming effects on several key defence proteins, leading to systemic acquired resistance to infection (Conrath et al., 2002). As we better understand these natural resilience mechanisms, more advanced treatments might be developed to mimic or activate these systemic resistance pathways to improve crop survival (Kohler et al., 2002), and some of these strategies may also be ‘borrowed’ for use in the clinic.

## Intersecting lessons

What this brief survey of repair mechanisms in plants and animals reveals is that there are indeed several strikingly analogous mechanisms, including ROS and calcium signalling, and metabolic alterations (Fig. 1). However, we highlight clear gaps in areas that are well studied in one kingdom but underexplored, and thus somewhat ‘under the radar’, in the other. For example, much is known about the role of AMPs in plant repair, but this has been studied to a much lesser extent in animal models besides *Drosophila* (Hanson and Lemaitre, 2020). The rise of antimicrobial resistance has created a demand for antimicrobials and pesticides in the clinic and in agriculture, and the cross-species application of AMPs could hold potential solutions for this crisis. An example of where cross-kingdom application is already happening is the plant defensin NmDef02, which has been recombinantly expressed in *Escherichia coli* and was shown to exhibit antimicrobial activity against human pathogens *in vitro*, as well as against plant pathogens (Ceballo et al., 2022). Similarly, the analogous resistance and resilience mechanisms, currently being uncovered in both plants and animals, can offer insights for research direction in diverse phyla. For example, ‘dampening’ mechanisms that limit cell death in plants may hold promise for novel therapeutics aiming to limit fibrosis in patients (Box 2). Now might be an ideal time for more systematic comparative studies on how plants and animals heal their wounds, and an opportunity for the two fields to learn from each other in ways that expand our options for novel treatments to enhance repair in the clinic and to replace pesticides and other agricultural treatments in crop production.
